# Terrestrial arthropods of Steel Creek, Buffalo National River, Arkansas. I. Select beetles (Coleoptera: Buprestidae, Carabidae, Cerambycidae, Curculionoidea excluding Scolytinae)

**DOI:** 10.3897/BDJ.3.e6832

**Published:** 2015-12-10

**Authors:** Michael Joseph Skvarla, Danielle M. Fisher, Kyle E. Schnepp, Ashley P.G. Dowling

**Affiliations:** ‡ University of Arkansas, Fayetteville, United States of America; § Florida State Collection of Arthropods, Gainesville, United States of America

**Keywords:** Anthribidae, Attelabidae, Brachyceridae, Brentidae, Buprestidae, Carabidae, Cerambycidae, Curculionidae, state record, range expansion, endemic, Interior Highlands, Boston Mountains

## Abstract

**Background:**

The Ozark Mountains are a region with high endemism and biodiversity, yet few invertebrate inventories have been made and few sites extensively studied. We surveyed a site near Steel Creek Campground, along the Buffalo National River in Arkansas, using twelve trap types – Malaise traps, canopy traps (upper and lower collector), Lindgren multifunnel traps (black, green, and purple), pan traps (blue, purple, red, white, and yellow), and pitfall traps – and Berlese-Tullgren extraction for eight and half months.

**New information:**

We provide collection records of beetle species belonging to eight families collected at the site. Thirty one species represent new state records: (Buprestidae) *Actenodes acornis*, *Agrilus cephalicus*, *Agrilus ohioensis*, *Agrilus paracelti*, *Taphrocerus nicolayi*; (Carabidae) *Agonum punctiforme*, *Synuchus impunctatus*; (Curculionidae) *Acalles clavatus*, *Acalles minutissimus*, *Acoptus suturalis*, *Anthonomus juniperinus*, *Anametis granulata*, *Idiostethus subcalvus*, *Eudociminus mannerheimii*, *Madarellus undulatus*, *Magdalis armicollis*, *Magdalis barbita*, *Mecinus pascuorum*, *Myrmex chevrolatii*, *Myrmex myrmex*, *Nicentrus lecontei*, *Otiorhynchus rugosostriatus*, *Piazorhinus pictus*, *Phyllotrox ferrugineus*, *Plocamus hispidulus*, *Pseudobaris nigrina*, *Pseudopentarthrum simplex*, *Rhinoncus pericarpius*, *Sitona lineatus*, *Stenoscelis brevis*, *Tomolips quericola*. Additionally, three endemic carabids, two of which are known only from the type series, were collected.

## Introduction

**Figure 1.**
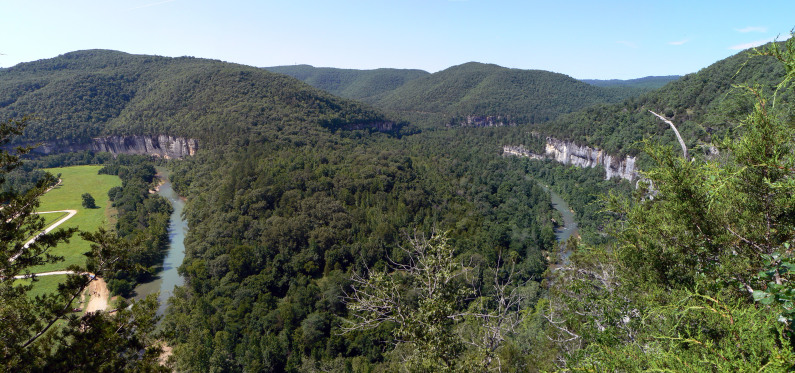
The Buffalo River from an overlook on the Buffalo River Trail near Steel Creek. Photo credit: Jasari. Used under Creative Commons license Attribution-ShareAlike 3.0 (CC BY-SA 3.0) (Creative Commons 2015).

**Figure 2.**
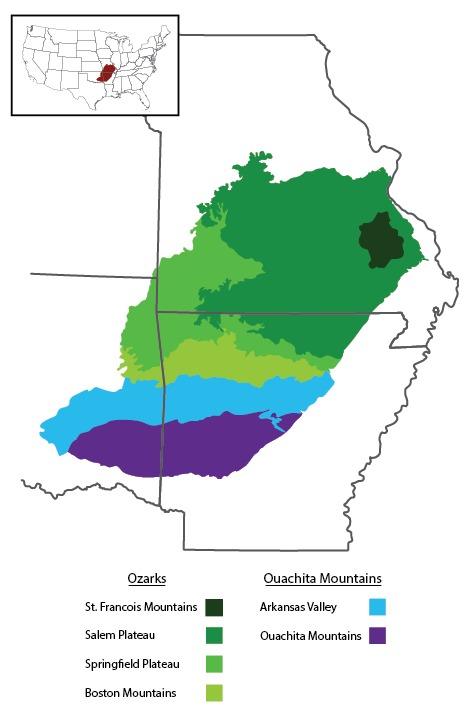
Geologic subregions of the Interior Highlands. Inset shows the region in context of the entire United States.

The Interior Highlands is a mountainous physiogeographic division in the central United States and the only significant topographic relief between the Appalachian and Rocky Mountains (Fig. 1). The area is known to harbor high biodiversity and many endemic species but remains grossly understudied. It is comprised of two regions with different geological histories: the Ouachita Mountains, which occupy west-central Arkansas and southeastern Oklahoma, and the Ozarks, which occupy southern Missouri, northern Arkansas, and extreme southeastern Kansas (Fig. 2).

The Ouachita Mountains are east-west trending fold mountains approximately 100 km wide and 190 km long (3,237,600 ha), with elevations up to 818 m (Robison and Allen 1995). They are the largest exposure of the Ouachita orogeny, which formed during the assembly of Pangea (by ~270 Ma); other exposures of the orogeny include the Marathon Mountains in Mexico and the base of the Sierra del Carmen in Coahuila, Mexico (Flawn 1968, Spearing 1991, U.S. Geological Survey 2014). Historically, the Ouachitas were connected to the Marathon Mountains to the west and Appalachian Mountains to the east. However, the break-up of Pangea and subsequent expansion of the Western Interior Seaway during the Cretaceous eroded and covered the mountains to the west while the formation of the Mississippi embayment, which resulted from the uplifting, rapid erosion, and subsequent subsidence of the area between the Ouachita and Appalachian Mountains from the mid-Cretaceous through early Cenozoic, severed the connection to the Appalachians (Carlton and Cox 1990, Spearing 1991, Cox and Van Arsdale 2002, Poole et al. 2005, U.S. Geological Survey 2014).

Prior to European settlement, the Ouachita Mountains were dominated by shortleaf pine (*Pinus echinata* Mill.), pine-hardwood, and mixed oak (*Quercus* L.) forests, with diverse, fire-dependent forb and grass understories (Hedrick et al. 1999); fire return intervals averaged 10 years and tree densities averaged 420 trees per ha with a mean diameter of 29 cm (Kreiter 1992, Masters et al. 1995). However, most virgin forest was heavily logged between 1910–1940 (Smith 1986) and presently tens of thousands of hectares have been converted to loblolly pine (*Pinus taeda* L.) plantations (Hedrick et al. 1999). The understory is dominated by woody vegetation and tree density has increased to 494–618 trees per ha while the mean diameter has decreased to 23 cm and average fire return intervals range from 40 to 1,200 years (Kreiter 1992, Masters et al. 1995).

The Ozarks, also referred to as the Ozark Mountains or Ozark Plateau, is divided into four geologic subdivisions. The Saint Francois Mountains, the oldest subdivision, is the exposed remains of a Proterozoic mountain range that formed through volcanic and intrusive activity 1485 Ma (Denison et al. 1984); it is also the smallest subdivision, covering approximately 180 square kilometers (Bretz 1965). The Salem Plateau, Springfield Plateau, and Boston Mountains are younger (Ordivician, Mississippian, and early Pennsylvanian age, respectively) plateaus that formed as the result of sedimentation and deposition along the edge of Laurentia. The Salem and Springfield Plateaus are composed largely of limestone and dolomite and are typified by karst topography, with thousands of caves and hundreds of springs documented in the region, while the Boston Mountains are composed largely of sandstone and shale (Bretz 1965, Arkansas Geological Survey 2015, Missouri Department of Natural Resources 2015, National Park Service 2015). The plateaus have been repeatedly uplifted and weathered, with the final uplift of the Ozarks occurring during the formation of the Ouachita orogeny; the region has remained exposed for the last 270 million years (Bretz 1965, Robison and Allen 1995, Guccione 2008, U.S. Geological Survey 2014).

The Salem and Springfield Plateaus rise to elevations of 450 m and 550 m, respectively, and are characterized by relatively flat plateau surfaces that form extensive plains cut into rolling, level-topped hills around rivers and other flowing water (Foti 2014). Oak/hickory forests and open woodlands are typical for the region, though extensive rocky, open glades can be common; additionally, the Springfield Plateau historically had extensive prairies, though these have largely been converted to agriculture (Foti 2014). The Boston Mountains is a highly dissected plateau, due to differential weathering of the relatively soft shale and harder sandstone, and the most rugged subdivision of the Ozarks, with an average elevation around 500 m and peaks up to 780 m. Oak/hickory forests predominate in most of the region, though drier south-facing slopes with extensive sandstone support short-leaf pine forests and moist, protected ravines support beech and sugar maple, which are uncommon elsewhere in the Ozarks (Foti 2014) For more information about the regions as they occur in Arkansas see Anderson 2006.

The Ouachita Mountains and Ozarks have never been connected as the Arkansas Valley (also called the Arkansas River Valley), which is part of the Arkoma Basin, formed as a foreland basin through downwarping along the Ouachita orogeny when the Ouachita Mountains were uplifted (Morris 1974, Wickham et al. 1976). The Arkansas River and its tributaries have increased the disconnection by eroding thousands of feet of sediment from the valley floor, which currently has an elevation of 90–150 m, and act as a physical barrier to poor-dispersing species (Carlton and Cox 1990, Foti and Bukenhofer 1998, Foti 2011). Differential erosion throughout the valley has left a few steep-sided, sandstone capped plateaus: Mount Magazine, Petit Jean Mountain, and Mount Nebo, which rise to elevations of 839 m, 741 m, and 411 m respectively (Higgins 2015, Peakery 2015).

The Interior Highlands can also be divided by ecoregion. Ecoregions, as defined by the Commission for Environmental Cooperation, are divided into three levels: Level I is the most inclusive and places the region "in context at global or intercontinental scales"; Level II regions are subdivisions of Level I regions and are "intended to provide a more detailed description of the large ecological areas nested within the level I regions"; finally, Level III has the smallest subdivisions that "enhance regional environmental monitoring, assessment and reporting, as well as decision-making" and "allow locally defining characteristics to be identified, and more specifically oriented management strategies to be formulated" (Commission for Environmental Cooperation 1997, Environmental Protection Agency 2015). At Level I, the Interior Highlands are included in the Eastern Temperate Forests, along with much of Eastern United States. At Level II the Interior Highlands are included in the Ozark, Ouachita-Appalachian Forests division, which also includes mountainous forests in the Appalachians. At Level III the Saint Francois Mountains, Salem and Springfield Plateaus are considered together as one subdivision – the Ozark Highlands – while the Boston Mountains, Arkansas Valley, and Ouachita Mountains are each considered separate subdivisions.

As may be expected with the regions inclusion in the Level I Eastern Temperate Forests ecoregion, many species found in the Interior Highlands are typical of eastern North America. However, some western species reach their eastern range limit in the Interior Highlands (e.g., Texas brown tarantula [*Aphonopelma hentzi* (Jean-Étienne Girard, 1852)], eastern collared lizard [*Crotaphytus collaris* (Say, 1823)], western diamondback rattlesnake [*Crotalus atrox* Baird & Girard, 1853]); these species likely colonized the Interior Highlands during the post-glacial Xerothermic Interval (6,000-4,000 b.p.), during which time prairies and xeric habitat similar to that in the west expanded into the Interior Highlands, and remained after the climate became more moist (Dowling 1956, Smith 1965, Trauth 1989, Trauth and Cochran 1992). Additionally, many species exhibit highly disjunct populations or are endemic to the region due to a number of factors: the abundance of caves and karst habitat support numerous localized cavernicolous species (Crandal 1998, Culver et al. 2000, Graening et al. 2003, Sarver and Lister 2004, Graening et al. 2006); rare habitats, such as xeric limestone prairies and glades, support specialized species assemblages (Baskin and Baskin 1988, Heikens 1999, Baskin and Baskin 2000, Ware 2002, Lawless 2005); previous connections to similar habitat (e.g., the Ouachitas and Appalachians, the River Valley plateaus and higher elevation habitat) have been severed for millions of years, allowing isolated populations of poor-dispersing organisms to speciate (e.g., Carlton and Cox 1990); and the Interior Highlands served as a refugia during periods of high sea levels and glaciation due to the unique geographic history discussed above (Redfearn 1986, The Nature Conservancy, Ozarks Ecoregional Assessment Team 2003).

The Nature Conservancy, Ozarks Ecoregional Assessment Team 2003 reported 58 species with highly disjunct populations in the Ozarks and a number of authors have discussed the disjunct populations of taxa in the region (birds: Selander 1965; fish: Bailey and Allum 1962; amphibians: Blair 1965; reptiles: Trauth et al. 2004; aquatic insects: Ross 1965; plants: Steyermark 1959, Redfearn 1986, Hemmerly 2002). While a comprehensive list of Interior Highland endemics is lacking, various authors have worked on geographic or taxonomic subsets: e.g., Pringle and Witsell 2005 stated that at least 20 species of plants are endemic to the Ouachita Mountains and Zollner et al. 2005 listed 36 plants endemic to the Interior Highlands; Allen 1990 reported 68 species of endemic insects and suggested there are at least 200 endemic plant and animal species in the Interior Highlands overall; Robison and Allen 1995 recorded 117 species endemic to Arkansas, most of which were found in the highland regions, though Robison et al. 2008 later reduced the number of Arkansas endemics to 100; and The Nature Conservancy, Ozarks Ecoregional Assessment Team 2003 reported 159 endemic species in the Ozarks. Additional disjunct and endemic species continue to be found and described (Table 1), so the number of such species is likely to continue to increase for the foreseeable future.

Aquatic insects and crayfish have been relatively well surveyed within the Interior Highlands (Table 2). Terrestrial insects and other arthropods, however, have been poorly surveyed and represent an excellent opportunity to find new endemic and disjunct species (though see Carlton and Robison 1998 concerning litter-dwelling beetles in the Ouachitas). This manuscript is the first in a series examining the arthropod fauna at a single site at Steel Creek along the Buffalo National River in the Boston Mountains of Arkansas. In addition to the new species records and other notes included below, it is intended to serve as an in-depth introduction and reference for future papers based on data collected during the study and other surveys in the Interior Highlands.

## Sampling methods

### Sampling description

The following traps were maintained within the site: five Malaise traps (MegaView Science Co., Ltd., Taichung, Taiwan), twenty-five pan traps (five of each color: blue, purple, red, yellow, white) which were randomly arranged under the Malaise traps (one of each color per Malaise trap) so as to also act as intercept traps; fifteen Lindgren multi-funnel traps (ChemTich International, S.A., Heredia, Costa Rica) (five of each color: black, green, purple); four SLAM (Sea, Land, and Air Malaise) traps (MegaView Science Co., Ltd., Taichung, Taiwan) with top and bottom collectors that acted as canopy traps; and seventeen pitfall trap sets. Sixteen of the seventeen pitfall sets were placed in two transects of sets spaced every five meters centered on two Malaise traps while the final set was placed away from other traps. Additionally, ten leaf litter samples were collected for Berlese extraction when traps were serviced.

Pitfall traps were based on a design proposed by Nordlander 1987; they were made using plastic soup containers and modified from the original design by cutting three slots into the side of each container instead of circular entrances. The slots were cut 2 cm under the rim and measured 2 cm tall x 9.3 cm wide, resulting in three equidistant 1.5 cm posts and a 28 cm collecting surface. The diameter at the base of the slots is approximately 10.5 cm and the cups are 10.5 cm deep below the slots, resulting in a collecting volume of 2,988 cm3. This design allowed the matching lids to be used as rain covers instead of using separate covers, such as ceramic tiles or bent metal sheeting. Each pitfall trap set was made by burying a single cup on either side of a 30.5 cm x 15.5 cm aluminum fence; trap catch from both cups was combined and treated as a single sample.

Berlese-Tullgren samples were collected from a variety of habitats, including thin leaf litter away from objects; thick leaf litter accumulated along logs and rocks; moss; tree holes; bark from fallen, partially decayed trees; and bark and leaf litter accumulated at the base of standing, dead trees. An attempt was made to collect moist, non-desiccated litter in order to increase the number of specimens collected; this resulted in fewer samples being taken from thin leaf litter, moss, and tree bark during the hot, dry summer months. Tree holes were sampled once each so as not to totally destroy them as potential habitat; as the number of tree holes within the site was limited, this resulted in only a handful of collections from this habitat type. Leaf litter samples were processed for four to seven days until the litter was thoroughly dry using modified Berlese-Tullgren funnels.

Trap placement began on 8 March 2013 and all traps were set by 13 March 2013, except Lindgren funnels, which were set on 1 April 2013. Traps set earlier than 13 March were reset on that date in order to standardize trap catch between traps. Traps were serviced approximately every two weeks (Table 3). The last collection of pitfall traps and pan traps occurred on 6 November 2013; Malaise, SLAM, and Lindgren funnel traps were run for an additional month, with the final collection on 4 December 2013. Berlese-Tullgren samples were not collected on 13 April, 15 May, and 6 November due to heavy rain that began during trap servicing and precluded sample collection. Berlese-Tullgren samples collected on 28 June were lost due to evaporation of ethanol in the funnel collecting cups after sample processing began. Pitfall cups were dislodged on 13 April (one set), 15 May (one set), 28 June (four sets), 17 July (five sets) due to unknown circumstances, though the pattern of litter and debris around the cups on two occasions suggested heavy rainfall and water accumulation forced the cups from the holes. In total, 1311 samples were collected (Table 4).

Propylene glycol (Peak RV & Marine Antifreeze) (Old World Industries, LLC, Northbrook, IL) was used as the preservative in all traps as it is non-toxic and generally preserves specimens well (Skvarla et al. 2014). Insect escape was impeded by the addition of a squirt of unscented, hypoallergenic dish detergent to the propylene glycol to act as a surfactant. Trap catch was sieved in the field and stored in Whirl-Pak bags (Nasco, Fort Atkinson, WI) in 90% ethanol until sorting.

### Quality control

Samples were coarse-sorted using a Leica MZ16 stereomicroscope illuminated with a Leica KL1500 LCD light source and a Wild M38 stereomicroscope illuminated with an Applied Scientific Devices Corp. Eco-light 20 fiber optic light source. After sorting, specimens were stored individually or by family in 2 mL microtubes (VWR International, LLC, Randor, PA) in 70% ethanol. Hard-bodied specimens (e.g., Carabidae, Curculionidae) were pinned or pointed as appropriate.

Specimens were identified with the use of published keys (Table 5).

The sole representative of *Lymantes* (Curculionidae) collected keys to *L. sandersoni* in Sleeper 1965. However, the character that separates *L. sandersoni* and *L. arkansasensis* is dubious, especially given that the two species are described from one and two specimens, respectively, from areas that are geographically similar and not widely separated (less than 300 km). Furthermore, R. S. Anderson, who is currently revising the genus, believes that all *Lymantes* in the eastern United States (excluding Texas) belong to a single species, *L. scrobicollis* (Paquin and Anderson 2009). Considering this, we identify the specimen collected as *L. sandersoni* with the caveat that it is likely that both *L. sandersoni* and *L. arkansasensis* will be synonymized with *L. scrobicollis* in the future.

*Ormiscus* (Curculionidae) consists of 14 described and approximately 30 undescribed species in North America north of Mexico (Valentine 2002). Species are most easily identified by the male secondary sexual features (e.g., characters on the mid and hind tibiae), however some species appear to be parthenogenetic (B. Valentine, pers. comm., via Iowa State University 2015), though this remains unconfirmed. In summary, this genus is in need of a major revision. As two-thirds or more of the North American species remain undescribed, we have declined to assign the single specimen collected to species.

Two weevil species, *Auleutes nebulosus* and *Laemosaccus nephele* (Curculionidae), are thought to be complexes of multiple cryptic species that are in need of revision (Anderson 2002, Ciegler 2010). As a limited number of specimens (2 and 4 per species complex, respectively) were collected, it is unlikely that multiple species were collected; additionally, modern revisions are lacking and identification of putative species is impossible. Specimens were therefore identified as the nominative species with the caveat that future studies may break the species complexes up and assign specimens collected in this study to other species.

The males of nine of 17 species of *Cercopeus* (Curculionidae) in the United States, including the widespread species *C. chrysorrhoeus*, are undescribed (O'Brien et al. 2010). All female *Cercopeus* collected in this study were identified as *C. chrysorrhoeus*; we therefore assumed that the males collected, which do not conform to the nine described males, are also *C. chrysorrhoeus*.

The *Chrysobothris femorata* (Buprestidae) species group consists of a dozen species that are difficult to seperate (with the exception of *C. adelpha*) as the characters used to distinguish species, including genitalia, are variable and often intermediate between species (Paiero et al. 2012). Further revision of the group is needed to positively identify species so, except for *C. adelpha*, we have chosen not to assign specimens to individual species.

All specimens have been deposited in the University of Arkansas Arthropod Museum (UAAM), with the following exceptions: 1) 1–5 exemplars of each species have been deposited in the Dowling Lab Collection at the University of Arkansas; 2) the following specimens were sent to Peter Messer for identification confirmation and have been deposited in the P. W. Messer Collection: *Agonum striatopunctatum* (MS 13-0529-072, #136215; MS 13-0612-022, #139663), *Cicindela rufiventris* (MS 13-0717-001, #134492), *Cyclotrachelus incisus* (MS 13-0413-023, #139591; MS 13-0413-019, #139592; MS 13-0413-006, #139594; MS 13-1008-075, #139596), *Cyclotrachelus parasodalis* (MS 13-0430-019, #131983; MS 13-0529-037, #135057; MS 13-1106-002, #138280), *Lophoglossus haldemanni* (MS 13-0529-066, #135053), *Pterostichus punctiventris* (MS 13-0401-018, #135065; MS 13-1023-021, # 136216), *Rhadine ozarkensis* (MS 13-0925-027, #134547), *Scaphinotus fissicollis* (MS 13-1106-037, #137830), *Selenophorus ellipticus* (MS 13-0925-005, #136223), *Selenophorus opalinus* (MS 13-0813-034, # 136217), *Trichotichus autumnalis* (MS 13-0730-005, #136226), *Trichotichnus vulpeculus* (MS 13-0911-027, #136218).

New Arkansas state records for Buprestidae are based on the range data given by Paiero et al. 2012; for Carabidae are based on range data given by Bousquet 2012b; and for Attelabidae and Curculionidae are based on O'Brien and Wibmer 1982 and supplemented by more recent literature (see individual species notes for specific citations). No attempt was made to assess the state record status of Cerambycidae as recent checklists and keys (e.g., Linsley 1962a, Linsley 1962b, Linsley 1963, Linsley 1964, Linsley and Chemsak 1972, Linsley and Chemsak 1976, Chemsak and Linsley 1982, Linsley and Chemsak 1984, Linsley and Chemsak 1995, Yanega 1996, Lingafelter 2007, Bezark and Monné 2013) report regional presence rather than presence by state and/or contain range maps for a few species with a limited number of records and J. A. Chemsak sadly passed before completing his "Illustrated Revision of the Cerambycidae of North America" series, which includes detailed range maps for the species treated (though see Chemsak 1996 for Parandrinae, Spondylidinae, Aseminae, and Prioninae and Chemsak 2007 for Lepturinae).

## Geographic coverage

### Description

The survey was conducted at 4 hectare plot established at Steel Creek along the Buffalo National River in Newton County, Arkansas, centered at approximately N 36°02.269', W 93°20.434'. The site is primarily 80–100 year old mature second-growth Eastern mixed deciduous forest dominated by oak (*Quercus*) and hickory (*Carya*), though American beech (*Fagus grandifolia*) and eastern red cedar (*Juniperus virginiana*) are also abundant. A small (14 m x 30 m), fishless pond and glade (10 m x 30 m) with sparse grasses are present within the boundaries of the site.

### Coordinates

36.0367 and 36.0397 Latitude; -93.3917 and -93.3397 Longitude.

## Taxonomic coverage

### Description

All specimens of Anthribidae, Attelabidae, Brachyceridae, Brentidae, Buprestidae, Carabidae, Cerambycidae, Curculionidae excluding Scolytinae were identified to species.

## Usage rights

### Use license

Creative Commons CCZero

## Data resources

### Data package title

Steel Creek survey

### Number of data sets

1

### Data set 1.

#### Data set name

Steel Creek beetles

#### Data format

Darwin Core Archive

#### Number of columns

34

#### Download URL

http://dx.doi.org/10.5061/dryad.4h40n

#### Data format version

1.0

#### Description

**Data set 1.** 

Column label	Column description
typeStatus	Nomenclatural type applied to the record
catalogNumber	Unique within-project and within-lab number applied to the record
recordedBy	Who recorded the record information
individualCount	The number of specimens contained within the record
lifeStage	Life stage of the specimens contained within the record
kingdom	Kingdom name
phylum	Phylum name
class	Class name
order	Order name
family	Family name
genus	Genus name
specificEpithet	Specific epithet
scientificNameAuthorship	Name of the author of the lowest taxon rank included in the record
scientificName	Complete scientific name including author and year
taxonRank	Lowest taxonomic rank of the record
country	Country in which the record was collected
countryCode	Two-letter country code
stateProvince	State in which the record was collected
county	County in which the record was collected
municipality	Closest municipality to where the record was collected
locality	Description of the specific locality where the record was collected
verbatimElevation	Average elevation of the field site in meters
verbatimCoordinates	Approximate center point coordinates of the field site in GPS coordinates
verbatiumLatitude	Approximate center point latitude of the field site in GPS coordinates
verbatimLongitude	Approximate center point longitude of the field site in GPS coordinates
decimalLatitude	Approximate center point latitude of the field site in decimal degrees
decimalLongitude	Approximate center point longitude of the field site in decimal degrees
georeferenceProtocol	Protocol by which the coordinates were taken
identifiedBy	Who identified the record
eventDate	Date or date range the record was collected
habitat	Description of the habitat
language	Two-letter abbreviation of the language in which the data and labels are recorded
institutionCode	Name of the institution where the specimens are deposited
basisofRecord	The specific nature of the record

## Additional information

### Analysis

8,048 specimens representing 251 species and 188 genera were collected during this study (Table 6), with the following totals by family: Anthribidae: 15 specimens, 4 species, 4 genera; Attelabidae: 19 specimens, 3 species, 3 genera; Brachyceridae: 1 specimen, 1 species, 1 genus; Brentidae: 6 specimens, 1 species, 1 genus; Buprestidae: 375 specimens, 27 species, 9 genera; Carabidae: 1970 specimens, 62 species, 36 genera; Cerambycidae: 1885 specimens, 82 species, 57 genera; Curculionidae: 3777 specimens, 71 species, 52 genera.

Thirty one species (12%) collected during this study represent new Arkansas state records: (Buprestidae) *Actenodes acornis*, *Agrilus cephalicus*, *Agrilus ohioensis*, *Agrilus paracelti*, *Taphrocerus nicolayi*; (Carabidae) *Agonum punctiforme*, *Synuchus impunctatus*; (Curculionidae) *Acalles clavatus*, *Acalles minutissimus*, *Acoptus suturalis*, *Anthonomus juniperinus*, *Anametis granulata*, *Eudociminus mannerheimii*, *Idiostethus subcalvus*, *Madarellus undulatus*, *Magdalis armicollis*, *Magdalis barbita*, *Mecinus pascuorum*, *Myrmex chevrolatii*, *Myrmex myrmex*, *Nicentrus lecontei*, *Otiorhynchus rugosostriatus*, *Piazorhinus pictus*, *Phyllotrox ferrugineus*, *Plocamus hispidulus*, *Pseudobaris nigrina*, *Pseudopentarthrum simplex*, *Rhinoncus pericarpius*, *Sitona lineatus*, *Stenoscelis brevis*, *Tomolips quericola*.

Three endemic carabids – *Cyclotrachelus parasodalis*, *Rhadine ozarkensis*, *Scaphinotus infletus* – were also collected.

### Notes on Select Species

*Agrilus ohioensis* (Buprestidae) has been recorded from many eastern states, but is rarely collected. Larvae have been reported from American hornbeam, *Carpinus caroliniana* Walter, (Nelson and MacRae 1990, Wellso and Jackman 2006) and winged elm, *Ulmus alata* Michx., (Nelson et al. 1981), both of which are present at the site. One reason for their apparent rarity may be from a lack of specialized collecting. Collecting small branches of hosts and rearing specimens is a specialized technique frequently used by wood borer enthusiasts. More work of this nature with these and other hosts should yield a wider distribution for this species and many other "rare" buprestids, including *Agrilus cephalicus*.

*Agonum punctiforme* (Carabidae) occurs from North Carolina to southeastern Texas, with a record from Missouri that "needs confirmed", and *Amara cupreolata* has been previously recorded in Arkansas but "the record needs confirmation" (Bousquet 2012b). It is thus unsurprising these species were collected in Arkansas.

**Figure 3.**
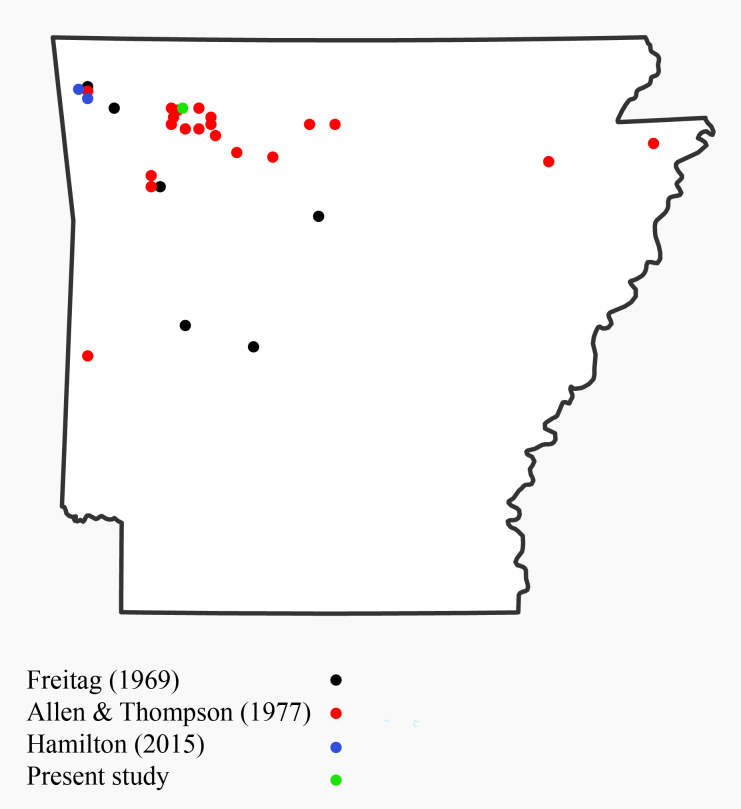
Known collection localities of *Cyclotrachelus parasodalis*.

*Cyclotrachelus parasodalis* (Carabidae) is an Arkansas endemic which has only been reported in the literature a handful of times, including the original description and description of the larvae (Freitag 1969, Allen and Thompson 1977, Thompson 1979, Hamilton 2015). Approximately 3,000 specimens are housed in the UAAM collection, most of which coincide with the collection localities and dates given by Allen and Thompson 1977, though the authors did not provide specific label data or the number of specimens collected per site in the publication (Fig. 3). Given the abundance of specimens and apparently wide range within the state, it is surprising the species has not been recorded in Missouri or Oklahoma sections of the Interior Highlands. Additionally, two specimens collected in cotton fields in the Mississippi Alluvial Plain indicate the species is not restricted entirely to the Interior Highlands, though it may be endemic to the region immediately surrounding the Interior Highlands.

*Rhadine ozarkensis* (Carabidae) is previously known only from the type series collected in Fincher’s Cave, near Black Oak, Arkansas (Washington County, not Craighead County) (Barr 1960, Bousquet 2012b). This specimen represents a range expansion of over 65 km. That it was collected in a pitfall trap on the surface suggests that the species may not be restricted to caves or can move between suitable cave habitat using the karst topography of the region.

*Pterostichus punctiventris* (Carabidae) ranges from northern Georgia south to Alabama west to east-central Missouri, eastern Oklahoma, and Texas (Bousquet 2012b). It is apparently known from a limited number of specimens and localities; in Arkansas, it has only been collected previously in Blanchard Springs State Park in Stone County (Bousquet 1992).

*Scaphinotus infletus* (Carabidae) is known from only three specimens collected from three localities within 30 km of the study site (Allen and Carlton 1988, Bousquet 2012b). This specimen represents a new locality for the species and confirms its presence in the area after nearly thirty years without being collected.

*Synuchus impunctatus* (Carabidae) is known from Missouri and Kansas, but has not previously been recorded from Arkansas (Bousquet 2012b).

*Tachys columbiensis* (Carabidae) was thought to be confined to the Coastal Plain and Piedmont Plateau, ranging from southeastern Pennsylvania to southern Florida west to Mississippi and eastern Texas, though it has also been recorded from central Arkansas (Pulaski and Garland Counties) (Bousquet 2012b). These specimens represent a new northwestern range limit and a new physiogeographic region (Ozark Mountains) for the species.

*Trichotichnus vulpeculus* (Carabidae) is recorded from western New Brunswick south to eastern Georgia, west to Wisconsin and northern Arkansas (Bousquet 2012b). These specimens are therefore likely near the southwestern range limit for this species.

*Acalles clavatus* (Curculionidae) was previously known from Florida, South Carolina and Louisiana (Ciegler 2010, O'Brien and Wibmer 1982); it has been reared from small twigs of *Quercus falcata* Michaux (Ferro et al. 2009).

*Acoptus suturalis* (Curculionidae) is known from northeastern North America, from Quebec south to North Carolina and Illinois and Iowa; addition records are known from Georgia and Mexico (O'Brien and Wibmer 1982). It has been raised from the branch of an American elm (*Ulmus americana* L.) and may be a vector of butternut canker virus (*Sirococcus clavigignenti-juglandacearum*) in butternut (*Juglans cinerea* L.) (Hoffman 1942, Halik and Bergdahl 2002).

*Anametis granulata* (Curculionidae) is found in northern and eastern North America, from Newfoundland and Quebec, south to New Jersey, west to Missouri, Wyoming and Montana; additional specimens are known from Texas, New Mexico, and Mexico (O'Brien and Wibmer 1982, Ocaña 1996).

*Anthonomus juniperinus* (Curculionidae) is known from the eastern United States, from Massachusetts south to Florida, west to West Virginia, as well as Texas, Oregon, and Paget, Bermuda (O'Brien and Wibmer 1982, Clark and Burke 2010). It feeds on *Gymnosporangium juniperi-virginianae* Schwein., a fungus parasitic on *Juniperus* L., and juniper berries (Ciegler 2010, Clark and Burke 2010).

*Buchananius sulcatus* (Curculionidae) is widely distributed in the eastern and southeastern United States (O'Brien and Wibmer 1982). It has been reared from the fruiting bodies of the ascomycete fungus *Trichoderma peltatum* (Berk.) Samuels, Jaklitsch, and Voglmayr (Prena et al. 2014) and adults have been collected in leaf litter and under branches (Kissinger 1957).

*Caulophilus dubius* (Curculionidae) is known from Quebec and New York south to Georgia, west to Illinois and and Mississippi, as well as Texas (O'Brien and Wibmer 1982, Douglas et al. 2013). Adults are found beneath dead tree bark and in tree holes (Blatchley and Leng 1916, Ciegler 2010).

*Eubulus bisignatus* (Curculionidae) is widespread in eastern and southern North America, ranging from Ontario south to Florida, west to Nebraska, Texas, Arizona, and California; it is also recorded from Mexico and Guatamala. It was not recorded from Arkansas by O'Brien and Wibmer 1982 but was reported by Anderson 2008. Adults are frequently collected at lights and in Malaise and flight-intercept traps and have been collected from a number of hardwood species including *Quercus* L., *Castanea* Mill., *Fagus* L., *Betula* L., *Carya* Nutt., and *Acer* L. (Anderson 2008.

*Eubulus obliquefasciatus* (Curculionidae) is commonly collected in flight-intercept traps and at lights. Adults have been collected on dead oak and sweetgum; otherwise, nothing is known about their biology (Anderson 2008).

The *Eudociminus mannerheimii* (Curculionidae) specimen collected during this study was included with other specimens collected near the field site in a forthcoming publication (Skvarla et al. in press) that suggests eastern red cedar (*Juniperus virginiana* L.) as a possible host as it is the only species of Cupressaceae present at the site. Additionally, the specimens represented a new state record and northwestern range expansion from previous records.

*Idiostethus subcalvus* (Curculionidae) is found from Pennsylvania south to South Carolina, west to Illinois and Missouri (O'Brien and Wibmer 1982, Ciegler 2010). Downie 1958 reported it is "very abundant" in April and May in Indiana. It been taken on *Caulophyllum thalictroides* (L.) Michaux, *Hydrophyllum appendiculatum* Michx., *Phacelia* Juss. and *Ranunculus hispidus* Michx. var. *nitidus* (Chapm.) T. Duncan (Robertson 1929, Ciegler 2010, Graham et al. 2012).

*Madarellus undulatus* (Curculionidae) is found in eastern North America, from Quebec and Connecticut south to Florida, west to South Dakota, Kansas, and Missouri (O'Brien and Wibmer 1982). It has been collected with black pyramid traps (Bloem et al. 2002), Malaise traps, fogging (Werle 2002) and at lights (Ciegler 2010). Larvae have been reported to feed on *Vitis* L., *Toxicodendron radicans* (L.) Kuntze and *Parthenocissus quinquefolia* (L.) Planch. (Blatchley and Leng 1916, Bouchard et al. 2005).

*Magdalis armicollis* (Curculionidae) is found in the eastern United States from Connecticut south to Georgia, west to North Dakota, Montana, Nebraska, and Texas (O'Brien and Wibmer 1982, Quinn 2000). Larvae mine galleries in stressed, dying, and dead *Ulmus* L. and adults feed on the leaves (Blatchley and Leng 1916, Hoffman 1942, Majka et al. 2007). Larval feeding is generally confined to branches smaller than 7.5 cm; however, in large numbers, larval and adult feeding can cause significant damage that may result in tree death (Baker 1941, Booth and Johnson 2009). *Magdalis armicollis* is not a vector of Dutch elm disease (Goeden and Norris 1963).

*Magdalis barbita* (Curculionidae) is found in North Ameica from Conneticut and Ontario south to Georgia, west to Montana, Texas, Nevada, and California (O'Brien and Wibmer 1982). Larvae mine galleries in the branches of dead and dying *Quercus*, *Ulmus*, and *Carya* and adults feed on the leaves of *Ulmus* (Blatchley and Leng 1916, Hoffman 1942, Majka et al. 2007). *Magdalis barbita* is not a vector of Dutch elm disease (Goeden and Norris 1963).

*Myrmex myrmex* (Curculionidae) is native to the eastern United States, from Conneticut south to Florida, west to Indiana and Iowa (O'Brien and Wibmer 1982). It develops in the dead and dying wood of sycamore (Burke et al. 1975), which was present in small numbers at the site.

*Notiodes limatulus* (Curculionidae) is widespread in North Ameica, ranging from New York south to Georgia, west to Idaho, Texas, and California, and into Mexico. It was not recorded in Arkansas by O'Brien and Wibmer 1982 but was reported in the state by O'Brien and Anderson 1996.

*Otiorhynchus rugosostriatus* (Curculionidae) is adventive from Europe and has been established in North America since 1876; it is now widespread through the United States and Canada (O'Brien and Wibmer 1982, Mattson et al. 1994). Larvae larvae feed on roots of Rosaceae and other plants (Mattson et al. 1994).

*Rhinoncus pericarpius* (Curculionidae) is adventive from the Palaerctic (Majka et al. 2007). It was first recorded in northeastern North America in 1895 and the Pacific Northwest in 1913; in the east it is known from Nova Scotia south through Georgia, west to Illinois (O'Brien and Wibmer 1982, Majka et al. 2007). *Rhinoncus pericarpius* is reported to feed on *Rumex* L. and *Cannabis* L. and have been collected from *Rheum* L. and *Medicago sativa* L. (Harada 1930, Hoebeke and Whitehead 1980).

*Stenoscelis brevis* (Curculionidae) is widespread is eastern North America, from Ontario and Quebec south to Florida, west to Wisconsin, Kansas, and Mississippi (O'Brien and Wibmer 1982). Larvae bore under the bark of dead hardwood (O'Brien 1997). Adults have been collected in Lindgren multifunnel traps baited with manuka oil, from leaf litter using Berlese extraction and under the bark of dead trees (Johnson et al. 2014, Ferro et al. 2012.

*Tachyerges niger* (Curculionidae) was not reported from Arkansas by O'Brien and Wibmer 1982 but was recorded from the state by Sweeney et al. 2012; it is assoxiated with *Salix* L.

*Tychius picirostris* (Curculionidae) is adventive from Europe and widely established in North America (Anderson and Howden 1994).

### Discussion

It is unsurprising that few Carabidae represented new state records as carabid workers formerly associated with the University of Arkansas (e.g., R. T. Allen, C. E. Carlton, R. G. Thompson) have thoroughly surveyed the region. Conversely, nearly one in five Buprestidae (19%) and one in three Curculionidae (32%) collected during this study represent new state records. Such high percentages of unrecorded species in charismatic and diverse taxa highlights how little attention many groups have received in the state and how much basic science and natural history is left to be done in 'The Natural State'.

Buprestids are capable of flying between habitat patches and rapidly colonizing new areas, so it is unlikely that new species will be discovered even though buprestids are understudied in the Interior Highlands. However, considering the high number of endemic species that are restricted to leaf litter habitats or are poor dispersers, how relatively understudied leaf litter weevils are, and that known but undescribed species were collected during this study, it is likely that the Interior Highlands is a fruitful area for finding new and disjunct weevil species.

## Figures and Tables

**Table 1.** Select references to recently discovered and described species with disjunct and endemic distributions in the Interior Highlands.

**Range status**	**Taxonomic category**	**Select references**
Disjunct	lichens	Lendenmer and Harris 2007, Harris and Ladd 2008, Harris and Lendemer 2009, Barton and Lendemer 2014, Lendemer and Harris 2014
	plants	Simurda and Knox 2000, Rimer and Summers 2006, Peck 2011
	molluscs	Nekola and Coles 2001
	arthropods	Carlton and Robison 1998
	fish	Berendzen et al. 2008
Endemic	lichens	Knudsen and Lendemer 2009
	plants	Rothrock and Reznicek 2001, Pringle and Witsell 2005, Campbell 2006, Nelson 2008, Floden et al. 2009, Yatskievych et al. 2013
	arthropods	Wolfe and Harp 2003, Sokolov et al. 2004, Holsinger et al. 2006, Dillman et al. 2010, Hildebrandt and Maddison 2011, Radwell et al. 2011
	fish	Kinzinger and Wood 2010, Adams et al. 2013

**Table 2.** Select references for well-sampled aquatic arthropods in the Interior Highlands.

**Taxon**	**Select references**
Ephemeroptera	McCafferty and Provonsha 1978, Sarver and Kondratieff 1997, Baumgardner and Kennedy 1999, Ferro and Sites 2007
Plecoptera	Ernst et al. 1986, Poulton and Steward 1991, Ferro and Sites 2007
Trichoptera	Bowles and Mathis 1989, Mathis and Bowles 1992, Moulton and Steward 1996, Ferro and Sites 2007, Etnier 2010
Astacoidea	Williams 1954

**Table 3.** Collection periods.

**Collection period**
13 March 2013 – 1 April 2013
1 April 2013 – 13 April 2013
30 April 2013 – 15 May 2013
15 May 2013 – 29 May 2013
29 May 2013 – 12 June 2013
12 June 2013 – 28 June 2013
28 June 2013 – 17 July 2013
17 July 2013 – 30 July 2013
30 July 2013 – 13 August 2013
13 August 2013 – 28 August 2013
28 August 2013 – 11 September 2013
11 September 2013 – 25 September 2013
25 September 2013 – 8 October 2013
8 October 2013 – 23 October 2013
23 October 2013 – 6 November 2013
6 Novemver 2013 – 20 November 2013
20 November 2013 – 4 December 2013

**Table 4.** Maximum number of traps collected (canopy, Lindgren funnel, Malaise, pan, and pitfall traps) or collections made (Berlese-Tullgren) per collecting period and total number of samples per sampling type; traps were occasionally destroyed or otherwise lost during the 2-week sampling period.

**Trap type**	**Number of traps or collections**	**Number of samples**
Berlese-Tullgren	10	140
Canopy trap (lower)	4	72
Canopy trap (upper)	4	72
Lindgren funnel (black)	5	85
Lindgren funnel (green)	5	85
Lindgren funnel (purple)	5	82
Malaise trap	5	95
Pan trap (blue)	5	82
Pan trap (purple)	5	81
Pan trap (red)	5	83
Pan trap (white)	5	83
Pan trap (yellow)	5	83
Pitfall	17	268

**Table 5.** References used for specimen identification.

**Family**	**Genus**	**Reference**
Anthribidae		Valentine 1998, Valentine 1960
Attelabidae		Hamilton 1971, Hamilton 1989, Hamilton 2002
Brentidae		Anderson and Kissinger 2002
Buprestidae		Nelson et al. 2008, Paiero et al. 2012
Carabidae		Lindroth 1969, Ciegler 2000, Arnett and Ivie 2001, Ball and Bousquet 2001, Pearson et al. 2006
Carabidae	* Abacidus *	Lindroth 1969, Sadek 1982
Carabidae	* Agonum *	Liebherr 1994
Carabidae	* Anisodactylus *	Noonan 1973
Carabidae	* Brachinus *	Erwin 1970
Carabidae	* Calathus *	Ball and Negre 1972
Carabidae	* Carabus *	Haldeman 1852
Carabidae	* Chlaenius *	Bell 1960
Carabidae	* Clinidium *	Bell and Bell 1975, Bell 1999
Carabidae	* Clivina *	Ball 2001, Bousquet 2009
Carabidae	* Cychrus *	Gidaspow 1973
Carabidae	* Cymindis *	Hunting 2013
Carabidae	* Dicaelus *	Ball 1959
Carabidae	* Dicheirus *	Noonan 1973
Carabidae	* Harpalus *	Noonan 1991
Carabidae	* Lebia *	Madge 1967
Carabidae	* Notiophilus *	Larochelle and Lariviere 1990
Carabidae	* Notobia *	Noonan 1973
Carabidae	* Platynus *	Liebherr and Will 1996, Bousquet 2012a
Carabidae	* Progaleritina *	Ball and Nimmo 1983
Carabidae	* Pseudophonus *	Ball and Anderson 1962
Carabidae	* Pterostichus *	Bousquet 1992
Carabidae	* Rhadinae *	Barr 1974
Carabidae	* Scaphinotus *	Van Dyke 1938, Allen and Carlton 1988
Carabidae	* Stenolophus *	Bousquet and Messer 2010
Carabidae	* Tachyta *	Erwin 1975
Cerambycidae		Yanega 1996, Lingafelter 2007
Cerambycidae	* Astylopsis *	Schiefer 2000
Cerambycidae	* Purpuricenus *	MacRae 2000
Cerambycidae	* Saperda *	Schiefer and Newell 2010
Curculionidae		Schaeffer 1907, Blatchley and Leng 1916, Anderson 2002, Hespenheide 2002, Ciegler 2010, Lyal 2010, WTaxa et al. 2012, Bright 1993, Bright and Bouchard 2008
Curculionidae	* Cercopeus *	O'Brien et al. 2010
Curculionidae	* Conotrachelus *	Schoof 1942
Curculionidae	* Cossonus *	Van Dyke 1915
Curculionidae	* Curculio *	Gibson 1969
Curculionidae	* Dichoxenus *	Sleeper 1956
Curculionidae	* Eubulus *	Anderson 2008
Curculionidae	* Geraeus *	Prena 2009
Curculionidae	* Lechriops *	Hespenheide 2003
Curculionidae	* Linogeraeus *	Prena 2009
Curculionidae	* Lissorhoptrus *	O'Brien and Haseeb 2014
Curculionidae	* Lymantes *	Sleeper 1965, Paquin and Anderson 2009
Curculionidae	* Notiodes *	Board 1972
Curculionidae	* Oopterinus *	O'Brien 1985
Curculionidae	* Otiorhynchus *	Warner and Negley 1976
Curculionidae	* Pandeletius *	Howden 1959
Curculionidae	* Rhinoncus *	Hoebeke and Whitehead 1980
Curculionidae	* Tychius *	Clark 1971
Curculionidae	* Tyloderma *	Wibmer 1918

**Table 6.** Species collected, including total number of specimens. New state records are indicated by an an asterisk (*).

*Family	**Genus**	**Species**	**Total specimens collected**
Anthribidae	* Euparius *	*Euparius marmoreus*	11
Anthribidae	* Eurymycter *	*Eurymycter fasciatus*	2
Anthribidae	* Ormiscus *		1
Anthribidae	* Toxonotus *	*Toxonotus cornutus*	1
Attelabidae	* Eugnamptus *	*Eugnamptus angustatus*	12
Attelabidae	* Synolabus *	*Synolabus bipustulatus*	1
Attelabidae	* Temnocerus *	*Temnocerus aeratus*	6
Brachyceridae	* Notiodes *	*Notiodes limatulus*	1
Brentidae	* Arrhenodes *	*Arrhenodes minutus*	6
Buprestidae	* Acmaeodera *	*Acmaeodera tubulus*	70
Buprestidae	* Acmaeodera *	*Acmaeodera pulchella*	1
Buprestidae	* Actenodes *	Actenodes acornis*	1
Buprestidae	* Agrilus *	*Agrilus arcuatus* complex	1
Buprestidae	* Agrilus *	*Agrilus bilineatus*	35
Buprestidae	* Agrilus *	Agrilus cephalicus*	18
Buprestidae	* Agrilus *	*Agrilus defectus*	1
Buprestidae	* Agrilus *	*Agrilus fallax*	1
Buprestidae	* Agrilus *	*Agrilus geminatus*	1
Buprestidae	* Agrilus *	*Agrilus lecontei*	4
Buprestidae	* Agrilus *	*Agrilus masculinus*	1
Buprestidae	* Agrilus *	Agrilus ohioensis*	1
Buprestidae	* Agrilus *	*Agrilus olentangyi*	1
Buprestidae	* Agrilus *	*Agrilus obsoletoguttatus*	12
Buprestidae	* Agrilus *	Agrilus paracelti*	3
Buprestidae	* Anthaxia *	*Anthaxia viridifrons*	6
Buprestidae	* Brachys *	*Brachys aerosus*	1
Buprestidae	* Chrysobothris *	*Chrysobothris adelpha*	60
Buprestidae	* Chrysobothris *	*Chrysobothris femorata* complex	70
Buprestidae	* Chrysobothris *	*Chrysobothris sexsignata*	7
Buprestidae	* Dicerca *	*Dicerca divaricata*	3
Buprestidae	* Dicerca *	*Dicerca lurida*	58
Buprestidae	* Dicerca *	*Dicerca obscura*	8
Buprestidae	* Dicerca *	*Dicerca spreta*	1
Buprestidae	* Ptosima *	*Ptosima gibbicollis*	5
Buprestidae	* Taphrocerus *	*Taphocerus gracilis*	3
Buprestidae	* Taphrocerus *	Taphrocerus nicolayi*	2
Carabidae	* Agonoleptus *	*Agonoleptus conjunctus*	17
Carabidae	* Agonum *	Agonum punctiforme*	2
Carabidae	* Agonum *	*Agonum striatopunctatum*	3
Carabidae	* Amara *	*Amara aenea*	3
Carabidae	* Amara *	*Amara cupreolata*	14
Carabidae	* Amara *	*Amara musculis*	30
Carabidae	* Anisodactylus *	*Anisodactylus rusticus*	33
Carabidae	* Apenes *	*Apenes sinuata*	8
Carabidae	* Badister *	*Badister notatus*	3
Carabidae	* Bembidion *	*Bembidion affine*	6
Carabidae	* Bembidion *	*Bembidion rapidum*	2
Carabidae	* Brachinus *	*Brachinus americanus*	91
Carabidae	* Calathus *	*Calathus opaculus*	14
Carabidae	* Calleida *	*Calleida viridipennis*	8
Carabidae	* Carabus *	*Carabus sylvosus*	20
Carabidae	* Chlaenius *	*Chlaenius platyderus*	1
Carabidae	* Chlaenius *	*Chlaenius tomentosus*	3
Carabidae	* Cicindela *	*Cicindela rufiventris*	3
Carabidae	* Cicindela *	*Cicindela sexguttata*	32
Carabidae	* Clinidium *	*Clinidium sculptile*	1
Carabidae	* Clivina *	*Clivina pallida*	1
Carabidae	* Cyclotrachelus *	*Cyclotrachelus incisus*	797
Carabidae	* Cyclotrachelus *	*Cylotrachelus parasodalis*	33
Carabidae	* Cymindis *	*Cymindis americana*	9
Carabidae	* Cymindis *	*Cymindis limbata*	203
Carabidae	* Cymindis *	*Cymindis platycollis*	8
Carabidae	* Dicaelus *	*Dicaelus ambiguus*	22
Carabidae	* Dicaelus *	*Dicaelus elongatus*	11
Carabidae	* Dicaelus *	*Dicaelus sculptilis*	78
Carabidae	* Dromius *	*Dromius piceus*	1
Carabidae	* Elaphropus *	*Elaphropus granarius*	1
Carabidae	* Galerita *	*Galerita bicolor*	19
Carabidae	* Galerita *	*Galerita janus*	2
Carabidae	* Harpalus *	*Harpalus faunus*	1
Carabidae	* Harpalus *	*Harpalus katiae*	1
Carabidae	* Harpalus *	*Harpalus pensylvanicus*	5
Carabidae	* Lebia *	*Lebia analis*	1
Carabidae	* Lebia *	*Lebia marginicollis*	1
Carabidae	* Lebia *	*Lebia viridis*	37
Carabidae	* Lophoglossus *	*Lophoglossus haldemanni*	1
Carabidae	* Mioptachys *	*Mioptachys flavicauda*	12
Carabidae	* Notiophilus *	*Notiophilus novemstriatus*	67
Carabidae	* Platynus *	*Platynus decentis*	9
Carabidae	* Platynus *	*Platynus parmarginatus*	2
Carabidae	* Plochionus *	*Plochionus timidus*	2
Carabidae	* Pterostichus *	*Pterostichus permundus*	105
Carabidae	* Pterostichus *	*Pterostichus punctiventris*	11
Carabidae	* Rhadine *	*Rhadine ozarkensis*	1
Carabidae	* Scaphinotus *	*Scaphinotus unicolor*	4
Carabidae	* Scaphinotus *	*Scaphinotus fissicollis*	12
Carabidae	* Scaphinotus *	*Scaphinotus infletus*	1
Carabidae	* Selenophorus *	*Selenophorus ellipticus*	4
Carabidae	* Selenophorus *	*Selenophorus gagatinus*	8
Carabidae	* Selenophorus *	*Selenophorus opalinus*	1
Carabidae	* Stenolophus *	*Stenolophus ochropezus*	5
Carabidae	* Synuchus *	Synuchus impunctatus*	3
Carabidae	* Tachyta *	*Tachyta parvicornis*	3
Carabidae	* Tachys *	*Tachys columbiensis*	4
Carabidae	* Tachys *	*Tachys oblitus*	2
Carabidae	* Trichotichnus *	*Trichotichnus autumnalis*	176
Carabidae	* Trichotichnus *	*Trichotichnus fulgens*	11
Carabidae	* Trichotichnus *	*Trichotichnus vulpeculus*	1
Cerambycidae	* Aegomorphus *	*Aegomorphus modestus*	8
Cerambycidae	* Aegormorphus *	*Aegormorphus quadrigibbus*	1
Cerambycidae	* Anelaphus *	*Anelaphus parallelus*	162
Cerambycidae	* Anelaphus *	*Anelaphus pumilus*	4
Cerambycidae	* Astyleiopus *	*Astyleiopus variegatus*	1
Cerambycidae	* Astylidius *	*Astylidius parvus*	2
Cerambycidae	* Astylopsis *	*Astylopsis macula*	4
Cerambycidae	* Astylopsis *	*Astylopsis sexguttata*	1
Cerambycidae	* Bellamira *	*Bellamira scalaris*	2
Cerambycidae	* Brachyleptura *	*Brachyleptura champlaini*	5
Cerambycidae	* Callimoxys *	*Callimoxys sanguinicollis*	4
Cerambycidae	* Centrodera *	*Centrodera sublineata*	1
Cerambycidae	* Clytoleptus *	*Clytoleptus albofasciatus*	6
Cerambycidae	* Cyrtinus *	*Cyrtinus pygmaeus*	5
Cerambycidae	* Cyrtophorus *	*Cyrtophorus verrucosus*	17
Cerambycidae	* Dorcaschema *	*Dorcaschema alternatum*	2
Cerambycidae	* Dorcaschema *	*Dorcaschema cinereum*	15
Cerambycidae	* Dorcaschema *	*Dorcaschema nigrum*	2
Cerambycidae	* Dorcaschema *	*Dorcaschema wildii*	2
Cerambycidae	* Eburia *	*Eburia quadrigeminata*	7
Cerambycidae	* Ecyrus *	*Ecyrus dasycerus*	1
Cerambycidae	* Elytrimitatrix *	*Elytrimitatrix undata*	30
Cerambycidae	* Elaphidion *	*Elaphidion mucronatum*	196
Cerambycidae	* Enaphalodes *	*Enaphalodes rufulus*	1
Cerambycidae	* Euderces *	*Euderces reichei*	1
Cerambycidae	* Euderces *	*Euderces picipes*	5
Cerambycidae	* Euderces *	*Euderces pini*	3
Cerambycidae	* Eupogonius *	*Eupogonius pauper*	2
Cerambycidae	* Gaurotes *	*Gaurotes cyanipennis*	1
Cerambycidae	* Graphisurus *	*Graphisurus despectus*	8
Cerambycidae	* Graphisurus *	*Graphisurus fasciatus*	10
Cerambycidae	* Heterachthes *	*Heterachthes quadrimaculatus*	18
Cerambycidae	* Hyperplatys *	*Hyperplatys maculata*	1
Cerambycidae	* Knulliana *	*Knulliana cincta*	10
Cerambycidae	* Leptostylus *	*Leptostylus transversus*	18
Cerambycidae	* Lepturges *	*Lepturges angulatus*	1
Cerambycidae	* Lepturges *	*Lepturges confluens*	9
Cerambycidae	* Micranoplium *	*Micranoplium unicolor*	3
Cerambycidae	* Molorchus *	*Molorchus bimaculatus*	65
Cerambycidae	* Monochamus *	*Monochamus titillator*	2
Cerambycidae	* Neoclytus *	*Neoclytus acuminatus*	60
Cerambycidae	* Neoclytus *	*Neoclytus caprea*	2
Cerambycidae	* Neoclytus *	*Neoclytus horridus*	2
Cerambycidae	* Neoclytus *	*Neoclytus jouteli*	1
Cerambycidae	* Neoclytus *	*Neoclytus mucronatus*	133
Cerambycidae	* Neoclytus *	*Neoclytus scutellaris*	129
Cerambycidae	* Necydalis *	*Necydalis mellita*	2
Cerambycidae	* Oberea *	*Oberea ulmicola*	1
Cerambycidae	* Obrium *	*Obrium maculatum*	10
Cerambycidae	* Oncideres *	*Oncideres cingulata*	2
Cerambycidae	* Orthosoma *	*Orthosoma brunneum*	7
Cerambycidae	* Parelaphidion *	*Parelaphidion aspersum*	7
Cerambycidae	* Phymatodes *	*Phymatodes amoenus*	2
Cerambycidae	* Phymatodes *	*Phymatodes testaceus*	8
Cerambycidae	* Phymatodes *	*Phymatodes varius*	4
Cerambycidae	* Physocnemum *	*Physocnemum brevilineum*	1
Cerambycidae	* Prionus *	*Prionus imbricornis*	1
Cerambycidae	* Purpuricenus *	*Purpuricenus humeralis*	1
Cerambycidae	* Purpuricenus *	*Purpuricenus paraxillaris*	13
Cerambycidae	* Saperda *	*Saperda discoidea*	9
Cerambycidae	* Saperda *	*Saperda imitans*	29
Cerambycidae	* Saperda *	*Saperda lateralis*	9
Cerambycidae	* Saperda *	*Saperda tridentata*	3
Cerambycidae	* Sarosesthes *	*Sarosesthes fulminans*	5
Cerambycidae	* Stenelytrana *	*Stenelytrana emarginata*	2
Cerambycidae	* Stenocorus *	*Stenocorus cinnamopterus*	7
Cerambycidae	* Stenosphenus *	*Stenosphenus notatus*	73
Cerambycidae	* Sternidius *	*Sternidius alpha*	6
Cerambycidae	* Strangalepta *	*Strangalepta abbreviata*	1
Cerambycidae	* Strangalia *	*Strangalia bicolor*	31
Cerambycidae	* Strangalia *	*Strangalia luteicornis*	205
Cerambycidae	* Strophiona *	*Strophiona nitens*	24
Cerambycidae	* Tilloclytus *	*Tilloclytus geminatus*	2
Cerambycidae	* Trachysida *	*Trachysida mutabilis*	2
Cerambycidae	* Trigonarthris *	*Trigonarthris minnesotana*	2
Cerambycidae	* Trigonarthris *	*Trigonarthris proxima*	3
Cerambycidae	* Typocerus *	*Typocerus lugubris*	2
Cerambycidae	* Typocerus *	*Typocerus velutinus*	46
Cerambycidae	* Typocerus *	*Typocerus zebra*	5
Cerambycidae	* Urgleptes *	*Urgleptes querci*	28
Cerambycidae	* Urgleptes *	*Urgleptes signatus*	9
Cerambycidae	* Xylotrechus *	*Xylotrechus colonus*	360
Curculionidae	* Acalles *	*Acalles carinatus*	11
Curculionidae	* Acalles *	Acalles clavatus*	5
Curculionidae	* Acalles *	Acalles minutissimus*	5
Curculionidae	* Acoptus *	Acoptus suturalis*	1
Curculionidae	* Anthonomus *	Anthonomus juniperinus*	1
Curculionidae	* Anthonomus *	*Anthonomus nigrinus*	3
Curculionidae	* Anthonomus *	*Anthonomus rufipennis*	5
Curculionidae	* Anthonomus *	*Anthonomus suturalis*	22
Curculionidae	* Aphanommata *	*Aphanommata tenuis*	9
Curculionidae	* Apteromechus *	*Apteromechus ferratus*	600
Curculionidae	* Anametis *	Anametis granulata*	5
Curculionidae	* Auleutes *	*Auleutes nebulosus* complex	2
Curculionidae	* Buchananius *	*Buchananius sulcatus*	4
Curculionidae	* Canistes *	*Canistes schusteri*	26
Curculionidae	* Caulophilus *	*Caulophilus dubius*	1
Curculionidae	* Cercopeus *	*Cercopeus chrysorrhoeus*	560
Curculionidae	* Chalcodermus *	*Chalcodermus inaequicollis*	1
Curculionidae	* Conotrachelus *	*Conotrachelus affinis*	9
Curculionidae	* Conotrachelus *	*Conotrachelus anaglypticus*	39
Curculionidae	* Conotrachelus *	*Conotrachelus aratus*	162
Curculionidae	* Conotrachelus *	*Conotrachelus carinifer*	56
Curculionidae	* Conotrachelus *	*Conotrachelus elegans*	44
Curculionidae	* Conotrachelus *	*Conotrachelus naso*	130
Curculionidae	* Conotrachelus *	*Conotrachelus posticatus*	979
Curculionidae	* Cophes *	*Cophes fallax*	73
Curculionidae	* Cophes *	*Cophes obtentus*	1
Curculionidae	* Cossonus *	*Cossonus impressifrons*	12
Curculionidae	* Craponius *	*Craponius inaequalis*	1
Curculionidae	* Cryptorhynchus *	*Cryptorhynchus fuscatus*	6
Curculionidae	* Cryptorhynchus *	*Cryptorhynchus tristis*	168
Curculionidae	* Curculio *	*Curculio othorhynchus*	1
Curculionidae	* Cyrtepistomus *	*Cyrtepistomus castaneus*	133
Curculionidae	* Dichoxenus *	*Dichoxenus setiger*	76
Curculionidae	* Dietzella *	*Dietzella zimmermanni*	1
Curculionidae	* Dryophthorus *	*Dryophthorus americanus*	30
Curculionidae	* Epacalles *	*Epacalles inflatus*	65
Curculionidae	* Eubulus *	*Eubulus bisignatus*	28
Curculionidae	* Eubulus *	*Eubulus obliquefasciatus*	193
Curculionidae	* Eudociminus *	Eudociminus mannerheimii*	1
Curculionidae	* Eurhoptus *	*Eurhoptus* sp. 1	28
Curculionidae	* Eurhoptus *	*Eurhoptus pyriformis*	15
Curculionidae	* Geraeus *	*Geraeus penicillus*	1
Curculionidae	* Hypera *	*Hypera compta*	4
Curculionidae	* Hypera *	*Hypera meles*	19
Curculionidae	* Hypera *	*Hypera nigrirostris*	1
Curculionidae	* Hypera *	*Hypera postica*	1
Curculionidae	* Idiostethus *	Idiostethus subcalvus*	1
Curculionidae	* Laemosaccus *	*Laemosaccus nephele* complex	3
Curculionidae	* Leichrops *	*Lechriops oculatus*	30
Curculionidae	* Lymantes *	*Lymantes sandersoni*	1
Curculionidae	* Madarellus *	Madarellus undulatus*	9
Curculionidae	* Magdalis *	Magdalis armicollis*	3
Curculionidae	* Magdalis *	Magdalis barbita*	5
Curculionidae	* Mecinus *	Mecinus pascuorum*	2
Curculionidae	* Myrmex *	Myrmex chevrolatii*	7
Curculionidae	* Myrmex *	Myrmex myrmex*	1
Curculionidae	* Nicentrus *	Nicentrus lecontei*	1
Curculionidae	* Oopterinus *	*Oopterinus perforatus*	17
Curculionidae	* Otiorhynchus *	Otiorhynchus rugosostriatus*	46
Curculionidae	* Pandeletius *	*Pandeletius hilaris*	51
Curculionidae	* Piazorhinus *	Piazorhinus pictus*	2
Curculionidae	* Phyllotrox *	Phyllotrox ferrugineus*	20
Curculionidae	* Plocamus *	Plocamus hispidulus*	1
Curculionidae	* Pseudobaris *	Pseudobaris nigrina*	9
Curculionidae	* Pseudopentarthrum *	Pseudopentarthrum simplex*	13
Curculionidae	* Rhinoncus *	Rhinoncus pericarpius*	1
Curculionidae	* Sitona *	Sitona lineatus*	1
Curculionidae	* Stenoscelis *	Stenoscelis brevis*	4
Curculionidae	* Tachyerges *	*Tachyerges niger*	1
Curculionidae	* Tomolips *	Tomolips quercicola*	2
Curculionidae	* Tychius *	*Tychius prolixus*	7
Curculionidae	* Tyloderma *	*Tyloderma foveolatum*	1
